# Distribution characteristics of human herpes viruses in the lower respiratory tract and their impact on 30-day mortality in community-acquired pneumonia patients

**DOI:** 10.3389/fcimb.2024.1436509

**Published:** 2024-08-16

**Authors:** Yadi Ding, Guiming Liu, Qiujing Li, Lingqing Zou, Jingyi Dai, Virasakdi Chongsuvivatwong

**Affiliations:** ^1^ Department of Public Laboratory, The Third People's Hospital of Kunming City, Infectious Disease Clinical Medical Center of Yunnan Province, Kunming, Yunnan, China; ^2^ International Research Fellow, Prince of Songkla University, Hat Yai, Songkhla, Thailand; ^3^ Epidemiology Unit, Faculty of Medicine, Prince of Songkla University, Hat Yai, Songkhla, Thailand

**Keywords:** community-acquired pneumonia, human herpes viruses, metagenomic next generation sequencing, human herpes virus 7, mortality

## Abstract

Human herpes viruses (HHVs) are commonly detected in community-acquired pneumonia (CAP) patients, particularly those with complex complications, attracting increased attention from clinical practitioners. However, the significance of detecting HHVs in bronchoalveolar lavage fluid (BALF) with CAP patients is still unclear. This study retrospectively analyzed BALF samples from 64 CAP patients at the Kunming Third People’s Hospital between August 2021 and December 2023. Metagenomic next generation sequencing (mNGS) was conducted on BALF samples during CAP onset. Multivariate Cox regression models were used to identify independent risk factors for 30-day all-cause mortality in CAP. HHVs were found in 84.4% of CAP patients, which were the most common pathogens (45.1%), followed by bacteria (30.2%) and fungi (11.5%). Bacterial-viral co-infections were most common, occurring in 39 patients. Notably, there was no significant difference in HHV presence between severe and non-severe CAP patients (EBV: *P* = 0.431, CMV: *P* = 0.825), except for HHV-7 (*P* = 0.025). In addition, there was no significant difference in the 30-day mortality between HHV positive and HHV negative groups (*P* = 0.470), as well as between the HHV-7 positive and HHV-7 negative groups (*P* = 0.910). However, neither HHVs nor HHV-7 was independent risk factors for 30-day mortality in CAP patients (HHVs: HR 1.171, *P* = 0.888; HHV-7: HR 1.947, *P* = 0.382). In summary, among the prevalent presence of multiple HHVs, EBV and CMV were the most prevalent in CAP patients. Patients with sCAP were more susceptible to HHV-7 than those with non-sCAP. These results provide valuable insights for clinicians in guiding appropriate interventions for CAP treatment.

## 1 Introduction

Community-acquired pneumonia (CAP) is a prevalent infection of the lower respiratory tract that has a significant effect on the health of adults, with an annual incidence ranging from 0.1% to 2.5% (Lu et al., 2023; Martin-Loeches et al., 2023). Notably, about 5.0-10.0% of CAP patients progress to severe community-acquired pneumonia (sCAP), leading to substantial morbidity, mortality, and economic costs (Liapikou, 2020; Niederman and Torres, 2022). This incidence increases with age, males, immunosuppressed status, and number of comorbidities (The Lancet Healthy Longevity, 2021; Niederman and Torres, 2022; Martin-Loeches et al., 2023).

Approximately 130 species of infectious herpesviridae have been identified, of which only eight are capable of infecting humans and known as human herpes viruses (HHVs) (Costa et al., 2019). HHVs including herpes simplex virus 1 (HSV-1), herpes simplex virus 2 (HSV-2), varicella-zoster virus (VZV), Epstein-Barr virus (EBV), human cytomegalovirus (CMV), human herpes virus 6 (HHV-6A, HHV-6B), human herpes virus 7 (HHV-7) and Kaposi’s sarcoma-associated herpes virus (KSHV) (Cohen, 2020; Connolly et al., 2021).

Human herpes viruses (HHVs) are widespread in CAP patients, once the host is infected, the virus can remain dormant in the host cells. Therefore, all HHVs can cause lifelong infection in the host, and cause disease when the virus is reactivated (Quach et al., 2023). Herpesviridae such as HSV-1, EBV, CMV and HHV-7 are commonly identified as primary pathogens in immunosuppressed patients or those with acute respiratory distress syndrome (ARDS) (Hraiech et al., 2019; Saura et al., 2022). HHVs had been extensively documented, which were associated with higher mortality in severe pneumonia patients (Huang et al., 2023; Liu et al., 2023b). HHV-7 has been observed in severe pneumonia patients, which were often in combination with co-infection of EBV and CMV (Xu et al., 2023). Additionally, studies have shown that EBV, CMV, HHV-7, and HHV-8 significantly increasing the risk of interstitial pneumonia or idiopathic pulmonary fibrosis (Sheng et al., 2020; De Rose et al., 2021). However, the significance of detecting HHVs in bronchoalveolar lavage fluid (BALF) from CAP patients remains unclear.

Due to the complex characteristics of CAP pathogens, there is an urgent need for metagenomic next generation sequencing (mNGS) as a rapid and effective diagnostic tool to identify pathogens in the lower respiratory tract, specifically in BALF. mNGS has proven to be more accurate and comprehensive compared to conventional detection methods, it can identify both known and unknown pathogens, leading to an improved detection rate and demonstrating its advantage in identifying multi-pathogenic CAP (Lv et al., 2023). This technology provides valuable insights to reduce delays in disease diagnosis and management (Sun et al., 2021; Lin et al., 2023).

The aim of this research was to improve the detection efficiency of pathogens in BALF by mNGS. Additionally, the study also aimed to assess the distribution characteristics of HHVs in CAP patients and their impact on 30-day mortality. Consequently, the findings offer important information for clinicians to guide timely interventions in treatment.

## 2 Materials and methods

### 2.1 Study population

This study retrospectively analyzed data from 82 patients admitted to Kunming Third People’s Hospital between August 2021 and December 2023. All patients had been diagnosed with CAP, and BALF samples were detected using mNGS within 24 hours of collection. Patients meeting the sCAP primary criteria or at least three sCAP secondary criteria were considered for potential admission to the intensive care unit (ICU) (Metlay et al., 2019).

An expert consensus on managing CAP in immunosuppressed patients was published in Chest 2020 (Ramirez et al., 2020). This study included immunosuppressed patients meeting the defined criteria. The exclusion criteria for the study were as follows: 1. Patients under 18 years of age, 2. Patients received mNGS after 30 days of admission, 3. Patients lost to follow-up, 4. Patients with incomplete clinical trial parameters.

### 2.2 Data collection

This study collected data from the electronic medical record system, which included demographic information such as gender, age, smoking history, drinking history, mechanical ventilation, length of stay (LOS), and underlying diseases. Additionally, various clinical trial parameters were collected 24 hours during the mNGS detection. These parameters included white blood cells, lymphocyte percentage, red blood cells, hematocrit, procalcitonin, hypersensitive C-reactive protein, bilirubin, urea, and creatinine. The severity and risk factors for death in CAP patients were assessed by calculating the SOFA score and the APACHE II score based on these parameters.

### 2.3 Statistical analysis

The Shapiro-Wilk test was used to assess the normality of the distribution of continuous variables. Variables that followed a normal distribution were represented by the means and standard deviations, while those not following a normal distribution were described using the median and quartiles. The data were classified utilizing either the χ2 test or the Fisher exact probability test. Frequency counts and percentages were used to summarize the results. Univariate comparisons between groups were conducted employing either the t-test or the Wilcoxon rank sum test for continuous variables. Kaplan-Meier analysis was used to compare the 30-day mortality of different groups after admission. Multivariate Cox regression models were utilized to identify independent risk factors for 30-day mortality, with continuous adjustment for variables. Cox.zph function were used to calculated the Schoenfeld residuals for each covariate to test the proportional hazards assumption of Cox regression. The hypothesis of parallelism was tested and the C-index was calculated. Sensitivity analysis was performed using different predictors in the Cox model to evaluate the robustness of the results. The statistical analysis and data visualization were performed using R software version 4.3.2. Significance levels were reported with double-tailed *P* values, with *P* < 0.05 deemed statistically significant. Pathogen stack was generated using Sangerbox3.0, while the pathogen proportion pie chart was created using SR plot (Tang et al., 2023).

## 3 Results

### 3.1 Clinical characteristics of CAP patients

**Figure 1:**
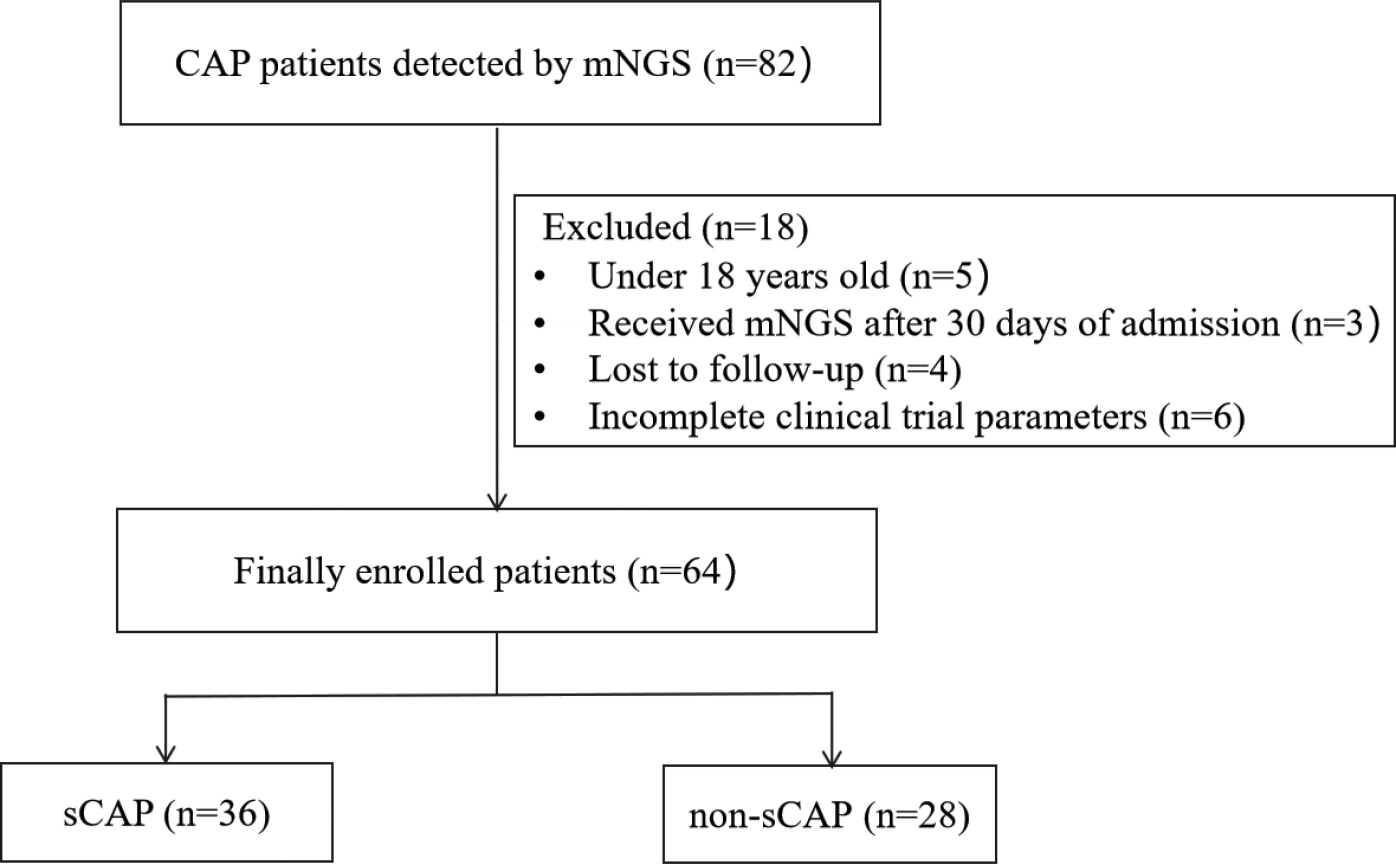
Flow chart of patients included in the study.

Initially, this study included a total of 82 CAP patients. Exclusions were made for patients under 18 years old (5 patients), those who received mNGS after 30 days of admission (3 patients), those lost to follow-up (4 patients), and those with incomplete clinical trial parameters (6 patients). The final analysis focused on 64 CAP patients, whose BALF samples were subjected to mNGS. Each patient was matched with one BALF sample. Based on the diagnostic criteria for sCAP, the CAP patients were divided into 36 sCAP patients and 28 non-sCAP patients (**Figure 1**).

In **Table 1**, it was observed that sCAP patients were older than non-sCAP patients (61.6 vs. 46.6, *P* = 0.002). Mechanical ventilation proved to be an effective respiratory support therapy for sCAP patients (72.2% vs. 25.0%, *P* < 0.001). The prevalence of underlying diseases such as respiratory insufficiency (*P* < 0.001), sepsis (*P* < 0.001), and hypertension (*P* < 0.001) was significantly higher in sCAP patients. While symptoms like fever, cough, and sputum are typically indicative of CAP, no significant contrast was observed between sCAP and non-sCAP patients in this regard. Clinical trial parameters varied significantly between the two groups (*P* < 0.001), including white blood cell count, lymphocyte percentages, procalcitonin levels, hypersensitive C-reactive protein levels, and urea levels. These parameters may help to distinguish patients who are not responding to treatment from those who are responding slowly, emphasizing the importance of closely monitoring sCAP patients. Moreover, the SOFA score (*P* < 0.001) and APACHE II score (*P* < 0.001) were notably higher in sCAP patients compared to non-sCAP patients.

**Table 1:** Baseline characteristics of 64 CAP patients.

Variables	Total (n=64)	sCAP (n=36)	non-sCAP (n=28)	*P* Value
Gender				0.705
Male, n (%)	45 (70.3)	26 (72.2)	19 (67.9)	
Female, n (%)	19 (29.7)	10 (27.8)	9 (32.1)	
Age, mean ± SD	55.1 ± 20.0	61.6 ± 18.2	46.6 ± 19.4	0.002
Smoking history, n (%)	24 (37.5)	15 (41.7)	9 (32.1)	0.435
Drinking history, n (%)	22 (34.4)	14 (38.9)	8 (28.6)	0.389
Mechanical Ventilation, n (%)	33 (51.6)	26 (72.2)	7 (25.0)	<0.001
Underlying disease				
Immunosuppressed status, n (%)	50 (78.1)	29 (80.6)	21 (75.0)	0.069
AIDS, n (%)	24 (37.5)	10 (27.8)	14 (50.0)	0.069
Renal disease, n (%)	18 (28.1)	13 (36.1)	5 (17.9)	0.107
Respiratory insufficiency, n (%)	35 (54.7)	32 (88.9)	3 (10.7)	<0.001
Sepsis, n (%)	21 (32.8)	19 (52.8)	2 (7.1)	<0.001
Diabetes-2, n (%)	13 (20.3)	10 (27.8)	3 (10.7)	0.092
Hypertension, n (%)	20 (31.2)	18 (50.0)	2 (7.1)	<0.001
Fever, n (%)	41 (64.1)	26 (72.2)	15 (53.6)	0.123
Cough, n (%)	54 (84.4)	33 (91.7)	21 (75.0)	0.090
Sputum, n (%)	50 (78.1)	31 (86.1)	19 (67.9)	0.080
Clinical trial parameters				
WBC (10^9/L, median [IQR])	7.8 (4.5, 11.7)	11.1 (7.7, 15.0)	4.6 (4.1, 6.8)	<0.001
LYMPH (%, median [IQR])	12.9 (6.9, 20.5)	7.8 (4.6, 14.7)	19.7 (12.0, 25.1)	<0.001
RBC (10^12/L, mean ± SD)	3.7 ± 0.9	3.7 ± 0.9	3.8 ± 0.9	0.456
HCT (%, mean ± SD)	33.9 ± 8.6	33.4 ± 8.9	34.5 ± 8.2	0.601
PCT (ng/ml, median [IQR])	0.2 (0.1, 1.1)	0.6 (0.1, 2.6)	0.1 (0.0, 0.2)	<0.001
hCRP (mg/L, median [IQR])	30 (11.2, 89.6)	71.4 (27.2, 103.2)	14.3 (5.2, 29.3)	<0.001
TBIL (umol/L, median [IQR])	8.2 (5.2, 13.8)	9.6 (6.0, 17.8)	7.7 (5.0, 11.2)	0.221
UREA (mmol/L, median [IQR])	5.3 (3.4, 8.6)	7.5 (5.7, 12.2)	3.6 (2.8, 4.7)	<0.001
CREA (umol/L, median [IQR])	63 (49.8, 79.2)	67.5 (52.8, 95.0)	56 (48.2, 66.0)	0.031
LOS (day, n (%))				0.594
<=14	14 (21.9)	7 (19.4)	7 (25.0)	
>14	50 (78.1)	29 (80.6)	21 (75.0)	
SOFA (score, median [IQR])	7 (4.8, 13.0)	11 (8.0, 16.0)	4 (3.8, 5.0)	<0.001
APACHE II (score, mean ± SD)	18.3 ± 9.1	23.7 ± 6.8	11.3 ± 6.8	<0.001

sCAP, severe community-acquired pneumonia; non-sCAP, non-severe community acquired pneumonia; AIDS, acquired immunodeficiency syndrome; WBC, white blood cells; LYMPH, lymphocyte percentage; RBC, red blood cells; HCT, hematocrit; PCT, procalcitonin; hCRP, hypersensitive C-reactive protein; TBIL, bilirubin; CREA, creatinine; LOS, length of stay; SOFA, sequential organ failure assessment; APACHE II, acute physiology and chronic health evaluation II.

### 3.2 Comparison of pathogens detected by mNGS between sCAP and non-sCAP groups

**Figure 2:**
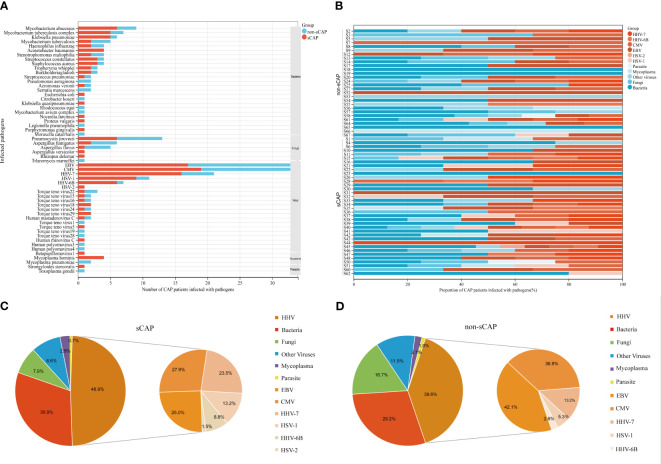
Compares and overlaps the pathogens in sCAP and non-sCAP groups. **(A)** Pathogens of lower respiratory tract infections in CAP patients. **(B)** Mixed infections in CAP patients. **(C, D)** The big pie chart on the left shows the distribution of pathogens detected by mNGS, and the small chart on the right shows the distribution of HHV detected by mNGS. **(C)** sCAP patients. **(D)** non-sCAP patients.

To further analyze the distribution characteristics of pathogens identified through mNGS, **Figure 2A** showed that 56 infectious pathogens were detected among the 64 CAP patients. These multiple pathogens included bacteria, fungi, viruses, mycoplasmas, and parasites. Specifically, 18 bacteria, 5 fungi, 16 viruses, 1 mycoplasma and 1 parasite were detected in sCAP patients, while 19 bacteria, 4 fungi, 15 viruses, 1 mycoplasma and 1 parasite were detected in non-sCAP patients. In addition, 12 bacteria, 3 fungi and 10 viruses were detected in both sCAP and non-sCAP patients. Notably, *Mycobacterium abscessus* (n=9) was the most common bacterial infection, *Pneumocystis jirovecii* (n=13) was the most common fungal infection, while EBV (n=33) and CMV (n=33) were the most common viral infections in CAP patients.

As shown in **Figure 2B**, co-infections were observed in 46 cases, with the remaining 18 cases associated with a single pathogen infection. The most common co-infections among the 64 CAP patients were bacterial-viral (39 cases), followed by fungal-viral (24 cases) and bacterial-fungal-viral (17 cases).

This study found that in 36 sCAP patients, the most prevalent pathogens were HHVs (48.9%), bacteria (30.9%), and fungi (7.9%), as shown in **Figure 2C**. Similarly, **Figure 2D** illustrated that in 28 non-sCAP patients, the most common pathogens were HHVs (39.6%), bacteria (29.2%), and fungi (16.7%). However, according to **Table 2**, no significant differences were observed between sCAP and non-sCAP patients in the presence of bacteria (*P* = 0.906), fungi (*P* = 0.309), HHVs (*P* = 0.090), other viruses (*P* = 0.622), mycoplasmas (*P* = 0.688) and parasites (*P* = 1.000).

**Table 2:** Comparison of pathogens detected by mNGS between sCAP and non-sCAP groups.

Infected pathogens	Total (n=64)	sCAP (n=36)	non-sCAP (n=28)	*P* Value
Bacteria, n (%)	41 (65.1)	23 (65.7)	18 (64.3)	0.906
Fungi, n (%)	23 (35.9)	11 (30.6)	12 (42.9)	0.309
HHVs, n (%)	54 (84.4)	33 (91.7)	21 (75.0)	0.090
EBV, n (%)	33 (51.6)	17 (47.2)	16 (57.1)	0.431
CMV, n (%)	33 (51.6)	19 (52.8)	14 (50.0)	0.825
HHV-7, n (%)	21 (32.8)	16 (44.4)	5 (17.9)	0.025
HSV-1, n (%)	11 (17.2)	9 (25.0)	2 (7.1)	0.013
HHV-6B, n (%)	7 (10.9)	6 (16.7)	1 (3.6)	0.271
HSV-2 *, n (%)	1 (1.6)	1 (2.8)	0 (0.0)	1.000
Other viruses, n (%)	23 (35.9)	12 (33.3)	11 (39.3)	0.622
Mycoplasma, n (%)	6 (9.4)	4 (11.1)	2 (7.1)	0.914
Parasite *, n (%)	2 (3.1)	1 (2.8)	1 (3.6)	1.000

*Fisher exact probability test was used for comparison between groups. χ^2^ test otherwise.

### 3.3 HHVs detected by mNGS

At least one HHV infection was detected in 84.4% of CAP patients. Among all pathogens detected, multiple HHVs were prevalent, including HSV-1, HSV-2, EBV, CMV, HHV-6B, and HHV-7. Only HSV-2 was detected in sCAP patients. There was no significant difference in HHVs between sCAP and non-sCAP patients (91.7% vs. 75.0%, *P* = 0.090) (**Table 2**).

Analysis in **Table 2** revealed that EBV (47.2% vs. 57.1%, *P* = 0.431) and CMV (52.8% vs. 50.0%, *P* = 0.825) were prevalent in both sCAP and non-sCAP patients, followed by HSV-1 and HHV-6B. The HHV-7 detection rate in sCAP patients was higher than that in non-sCAP patients (44.4% vs. 17.9%, *P* = 0.025).

### 3.4 Relationship between HHVs and 30-day mortality in CAP patients

**Figure 3:**
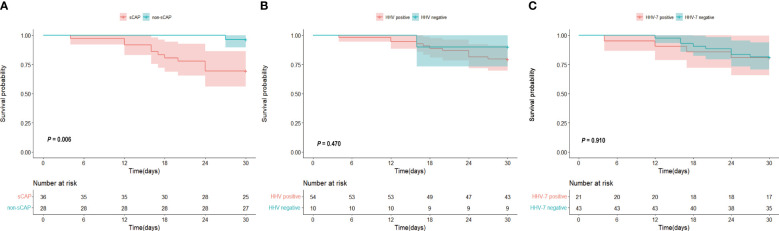
**(A–C)** Kaplan-Meier curve 30 days of **(A)** sCAP and non-sCAP groups, **(B)** HHV positive and HHV negative groups, **(C)** HHV-7 positive and HHV-7 negative groups.

To investigate the 30-day mortality of different groups, we plotted a survival curve. **Figure 3A** revealed a significantly higher 30-day mortality in sCAP patients compared to non-sCAP patients. Specifically, the 30-day mortality was 30.6% in sCAP patients, whereas it was only 3.6% in non-sCAP patients. In **Figure 3B**, although there was no statistically significant difference in 30-day mortality between HHV positive and negative groups (*P* = 0.470), the 30-day mortality was higher in the HHV positive group. Finally, **Figure 3C** showed no significant variance in the 30-day mortality between HHV-7 positive and HHV-7 negative groups (*P* = 0.910), which may have been influenced by the fact that there were fewer patients in the HHV-7 positive group than in the HHV-7 negative groups.

### 3.5 All-cause 30-day mortality

**Figure 4:**
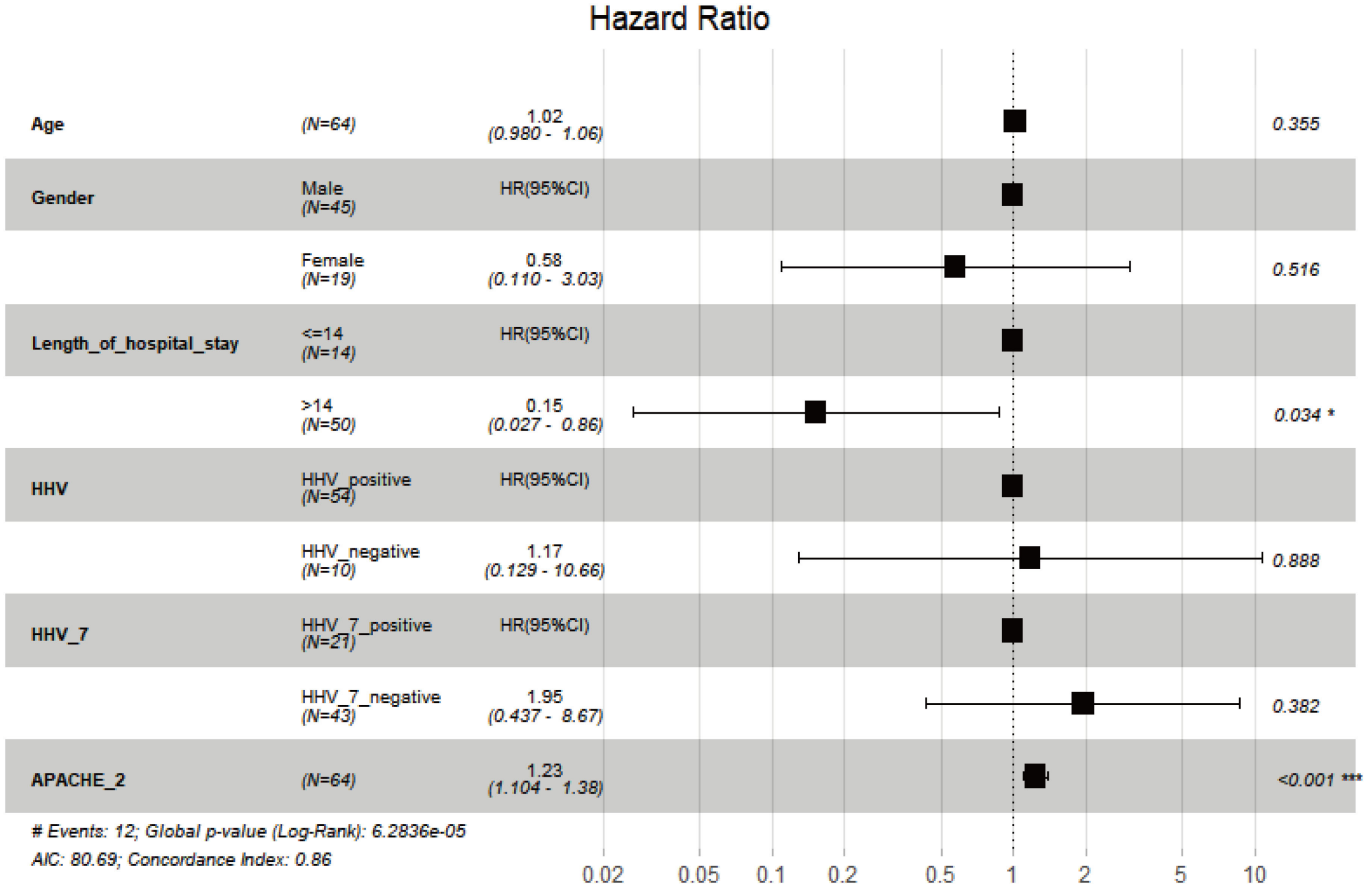
Multivariate analysis of 30-day all-cause mortality of CAP using Cox regression model. *, *P* < 0.05; ***, *P* < 0.001.

A Cox multivariate regression analysis was conducted to investigate the influence of gender, age, LOS, HHVs, HHV-7, and APACHE II score on the 30-day mortality in CAP patients. The results indicated that neither HHVs (HR 1.171, *P* = 0.888) nor HHV-7 (HR 1.947, *P* = 0.382) was independent risk factors for 30-day mortality in CAP patients (**Figure 4**).

The proportional hazards assumption for Cox regression was evaluated. The *P* values for both the individual variable test and the global variable test were all greater than 0.05, indicating that the proportional hazards assumption was not violated. We calculated the Harrell’s C-index (0.791), indicating model fitting is better. Additionally, we performed a sensitivity analysis on the Cox regression model, and we observed that the hazard ratio (HR) value for HHV-7 remained largely unchanged even when other covariates were added or modified.

## 4 Discussion

This study discovered that the older individuals were more vulnerable to sCAP, which aligns with previous research (Lin et al., 2023). The global issue of aging is becoming increasingly critical (Covino et al., 2020; Fan et al., 2024). CAP has emerged as a significant disease affecting individuals aged 65 and above, posing a serious health risk and a major public health concern (Arnold et al., 2020). Consequently, enhancing the awareness about CAP among older individuals, especially in sCAP cases, and prioritizing comprehensive treatment and quality care are crucial for improving the prognosis of CAP in this demographic (Li and Chu, 2023).

HHVs were the predominant infection in CAP patients, followed by bacteria and fungi. Interestingly, the number of viral pathogens detected in this study exceeded the combined number of bacterial and fungal pathogens, reflecting a similar trend observed in CAP patients admitted to hospitals in the United States (Jain et al., 2015). Among the HHVs, EBV and CMV were the most common viruses causing lower respiratory tract infections in CAP patients, which aligned with the findings of a previous multi-center retrospective study (Huang et al., 2023). These findings indicated the accuracy of our CAP pathogen detection rates.

The most prevalent bacterial infection in the lower respiratory tract was *Mycobacterium abscessus*, with *Pneumocystis jirovecii* being the most common fungal infection, consistent with previous research (Wei et al., 2020; Wu et al., 2020; Schwartz et al., 2023). Previous studies have identified *Streptococcus pneumoniae* as the main pathogen causing CAP (Jain et al., 2015; Ferrer et al., 2018; Liu et al., 2023a). It is important to highlight that these studies excluded immunosuppressed patients and did not use mNGS to detect BALF samples, making comparisons with our study challenging. Our research focused on CAP patients from infectious disease hospitals, considering complex complications like immunosuppression, AIDS, renal disease, respiratory insufficiency, and sepsis. Notably, these complications can impact the infectious pathogens. This may be related to the fact that after HHVs infection, the virus replicates and spreads in the body, leading to a decline in the immune system, which increases susceptibility to other pathogens. Immunosuppression is a common risk factor for CAP (Brands et al., 2020), which is why we intentionally included immunosuppressed patients in our study. Interestingly, we did not find a significant difference between sCAP and non-sCAP patients, in line with previous research on CAP caused by respiratory syncytial virus (Di Pasquale et al., 2019; Bahabri et al., 2023).

Patients with sCAP were more susceptible to HHV-7 than those with non-sCAP. Nonetheless, HHV-7 did not independently contribute to the risk of 30-day mortality, as observed in other severe pneumonia cases (Xu et al., 2023). This may be due to the fact that HHV-7 infection is asymptomatic in most adults, so its direct lethality may be relatively low. While a vaccine exists for varicella-zoster virus (VZV) (Curtis et al., 2021), there is currently no efficient method for preventing and treating HHV-7. Nucleoside analogues like acyclovir, ganciclovir, and valaciclovir may be utilized, although ganciclovir proves to be less effective against HHV-7 in comparison to HHV-6 and CMV (Ärlemalm et al., 2022; Sureram et al., 2022). Notably, the presence of HHV-7 in sCAP may potentially exacerbate lung inflammation and systemic inflammatory responses in conjunction with other viruses and bacteria. Thus, it is imperative to investigate whether HHV-7 poses a pathogenic threat to sCAP patients, emphasizing the necessity for the development of targeted anti-HHV medications and safe, effective vaccines. Targeted anti-infective therapy for individuals infected with HHVs play a crucial role in preventing recurrence.

There were several limitations to our research that must be recognized. Firstly, the follow-up period for mortality was limited to 30 days in our study, which was consistent with existing research (Simonetti et al., 2016; Jones et al., 2020). Given that CAP as a lung parenchymal infection, almost all patients showed HHVs by day 28 (Ong et al., 2017). There was a possibility that patients with COVID-19 pneumonia had HSV-1 reactivation within 30 days in BALF samples (Giacobbe et al., 2022). Current international guidelines stressed the importance of prioritizing the 30-day mortality outcome in studies (Martin-Loeches et al., 2023). Secondly, our study was cross-sectional, and mNGS detection of BALF samples was only conducted at the onset of CAP. Therefore, our results can only reflect the characteristics of HHVs distribution during this specific period and can not be generalized to the entire of CAP. Previous researches have shown that HHVs significantly increased with the length of hospital stay (Ong et al., 2017; Huang et al., 2023). Lastly,our study was a single retrospective analysis with a limited sample size, which impeded the generalizability of our findings. Nonetheless, it is crucial to emphasize that the application of mNGS technology facilitated the identification of all pathogens in CAP patients objectively, ensuring a uniform approach to the diagnosis and treatment of mixed infections.

## 5 Conclusion

Among the prevalent presence of multiple HHVs, EBV and CMV were the most prevalent in CAP patient. Patients with sCAP were more susceptible to HHV-7 than those with non-sCAP. However, neither HHVs nor HHV-7 was independent risk factors for 30-day mortality in CAP patients. The findings offer important information for clinicians to guide timely interventions in treatment.

## Data availability statement

The sequencing raw data for this study is public and can be found here: NCBI SRA under project ID: PRJNA1131635.

## Ethics statement

The studies involving humans were approved by Ethics Committee of Kunming Third People’s Hospital (No. KSLL20230711009). The studies were conducted in accordance with the local legislation and institutional requirements. Written informed consent for participation was not required from the participants or the participants’ legal guardians/next of kin in accordance with the national legislation and institutional requirements.

## Author contributions

YD: Data curation, Formal analysis, Methodology, Resources, Writing – original draft, Writing – review & editing. GL: Data curation, Formal analysis, Investigation, Writing – original draft, Writing – review & editing. QL: Data curation, Investigation, Resources, Writing – original draft, Writing – review & editing. LZ: Data curation, Formal analysis, Methodology, Software, Writing – review & editing. JD: Supervision, Validation, Writing – original draft, Writing – review & editing. VC: Supervision, Visualization, Writing – original draft, Writing – review & editing.
