# Corrigendum: Distribution characteristics of human herpes viruses in the lower respiratory tract and their impact on 30-day mortality in community-acquired pneumonia patients

**DOI:** 10.3389/fcimb.2024.1488546

**Published:** 2024-09-17

**Authors:** Yadi Ding, Guiming Liu, Qiujing Li, Lingqing Zou, Jingyi Dai, Virasakdi Chongsuvivatwong

**Affiliations:** ^1^ Department of Public Laboratory, The Third People’s Hospital of Kunming City, Infectious Disease Clinical Medical Center of Yunnan Province, Kunming, Yunnan, China; ^2^ International Research Fellow, Prince of Songkla University, Hat Yai, Songkhla, Thailand; ^3^ Epidemiology Unit, Faculty of Medicine, Prince of Songkla University, Hat Yai, Songkhla, Thailand

**Keywords:** community-acquired pneumonia, human herpes viruses, metagenomic next generation sequencing, human herpes virus 7, mortality

In the published article, there was an error in the **Funding** statement. “This research was supported by a postdoctoral fellowship from Prince of Songkla University, Hat Yai, Thailand, and supported by Kunming Science and Technology Bureau (2023-1-NS-007)”. The correct **Funding** statement appears below.

“This research was financially supported by Prince of Songkla University and Ministry of Higher Education, Science, Research and Innovation under the Reinventing University Project (Grant Number REV65022), and Kunming Science and Technology Bureau (2023-1-NS-007)”.

The authors apologize for this error and state that this does not change the scientific conclusions of the article in any way. The original article has been updated.

